# An online escape room as an icebreaker in interdisciplinary and international health care professions education: A pilot mixed-methods study of “UKA Escape”

**DOI:** 10.3205/zma001742

**Published:** 2025-04-15

**Authors:** Martin Lemos, Veronique Kouchev, Laura Bell

**Affiliations:** 1 RWTH Aachen University, Medical Faculty, Audiovisual Media Center, Aachen, Germany

**Keywords:** online escape room, health professions education, team-based learning, icebreaker activity

## Abstract

This pilot study explored the use of an online escape room at RWTH Aachen University Hospital (UKA Escape) as an icebreaker. The game was developed for the introductory day of an international program aimed at teaching scientific skills in health professions education. The group activity in the online escape room was designed to provide an engaging introduction to the course and the subsequent learning group(s). Based on the story of a medical researcher and her team, who have developed a revolutionary serum, students must try to escape from the digital lab and save the serum. To this end, the students worked in five small groups to solve science-related puzzles. An interactive and synchronous collaboration was facilitated despite physical distance using a video conferencing tool.

The usability of the digital escape room was rated in the fourth quartile (system usability scale score of *M*=81.25). In line with the questionnaire-based feedback, students’ qualitative feedback was predominantly positive, with indications of areas for improvement. Overall, the results indicate that the students greatly appreciated the online escape room and that teamwork was encouraged, demonstrating the potential of escape rooms in health professions education.

## Abstract (de)

Diese Pilotstudie untersuchte den Einsatz eines Online-Escape-Rooms an der Uniklinik RWTH Aachen (UKA Escape) als Icebreaker. Das Spiel wurde für den Einführungstag eines internationalen Programms entwickelt, das auf die Vermittlung wissenschaftlicher Fähigkeiten in der Ausbildung von Gesundheitsberufen abzielt. Die Gruppenaktivität im Online-Escape-Room soll als ein ansprechender Einstieg in den Kurs dienen und eine Grundlage für die Zusammenarbeit in der Lerngruppe schaffen. Basierend auf der Geschichte einer medizinischen Forscherin und ihres Teams, die ein revolutionäres Serum entwickelt haben, müssen die Studierenden versuchen, aus dem digitalen Labor zu entkommen und das Serum zu retten. Zu diesem Zweck arbeiteten die Studierenden in fünf Kleingruppen, um wissenschaftsbezogene Rätsel zu lösen. Die interaktive und synchrone Zusammenarbeit wurde trotz der räumlichen Entfernung durch ein Videokonferenz-Tool ermöglicht.

Die Benutzerfreundlichkeit des Online-Escape-Rooms wurde im vierten Quartil bewertet (System Usability Scale Score von *M*=81,25). In Übereinstimmung mit dem fragebogenbasierten Feedback war das qualitative Feedback der Studierenden überwiegend positiv, mit Hinweisen auf verbesserungswürdige Bereiche. Insgesamt deuten die Ergebnisse darauf hin, dass die Studierenden den Online-Escape-Room sehr schätzten und die Teamarbeit gefördert wurde, was auf das generelle Potenzial von Escape-Rooms in der Ausbildung von Gesundheitsberufen hinweist.

## Introduction

The use of escape rooms in health care professions education (HPE) has gained attention as a potential tool to engage students and facilitate active learning [1], [2], [3]. 

An (online) escape room (OnER) is an immersive, themed experience set within a confined space, often intricately designed to emulate specific environments or scenarios. In this setting, participants are usually grouped into teams and given a fixed time frame to decipher a series of puzzles, riddles, and challenges. The goal is to complete interconnected tasks that lead to predetermined objectives and enable participants to “escape” the room. Escape rooms offer a unique, immersive experience that actively engages participants in problem-solving, critical thinking, and teamwork [4]. Effective teamwork, communication as well as group cohesion, i.e. the sense of involvement and affiliation, are shown to be among six conceptual categories crucial for effective (interdisciplinary) teams [5]. Furthermore, escape rooms allow students to apply their knowledge practically and interactively [6], [7], [8]. Ross et al. [1] described the use of an OnER format in online nutrition and dietetics education as an innovative and effective learning strategy, receiving overwhelmingly positive feedback from students. As reported by Hursman et al. [8], there was an increase in the team’s confidence in accomplishing tasks, effective group communication, and a heightened perception of the importance of teamwork after using OnER. However, the use and benefit of OnER have not yet been studied in a geographically heterogeneous and interdisciplinary health care profession population. The current study, therefore, investigated whether integrating an OnER into an international HPE curriculum could enhance student engagement and help break the ice within small groups.

## Project description

A pilot study was conducted using a mixed-methods and a quasi-experimental design during the welcome day of an international program for learning scientific skills in HPE (SEMERA [https://semera.rwth-aachen.de/]) at the RWTH Aachen University. The number of participants in the program is currently limited to 35 participants. Designed to promote initial student engagement, the OnER aimed to break the ice within the study groups and familiarize them with the program content and the setting of the RWTH Aachen University Hospital. The study goal was to evaluate the usability and acceptance of OnER and to ascertain whether, from the students’ perspective, OnER helps teams work together and interact with each other.

The activity was planned considering that health care profession students would join from various locations across different geographical regions. To bridge the physical gaps and foster a cohesive learning environment, the video conferencing platform Zoom [https://zoom.us] was used. This platform enabled interaction and collaboration between the participants in the small groups in real-time. The 35 students were assigned to five groups, with seven students each, by the SEMERA coordination for the whole program, considering nationality, gender, and field of study. For the OnER, students were split into breakout rooms, and one student shared the screen with the game to facilitate synchronization of the activities and group discussions.

The “UKA escape” main storyline starts with the following introduction: “Dr Irene Hoffmann, a brilliant medical researcher, and her team have developed a revolutionary serum that has the potential to save millions of lives. But shortly before the official announcement of her discovery, she disappears without a trace. You and your team are sent to her private research lab at the RWTH Aachen University Hospital to find and secure the serum. But as you enter the lab, the door slams shut behind you and a timer is activated: You have exactly 15 minutes to find the code for the door and escape before the entire lab is sealed forever – with you inside!”

**Figure 1:**
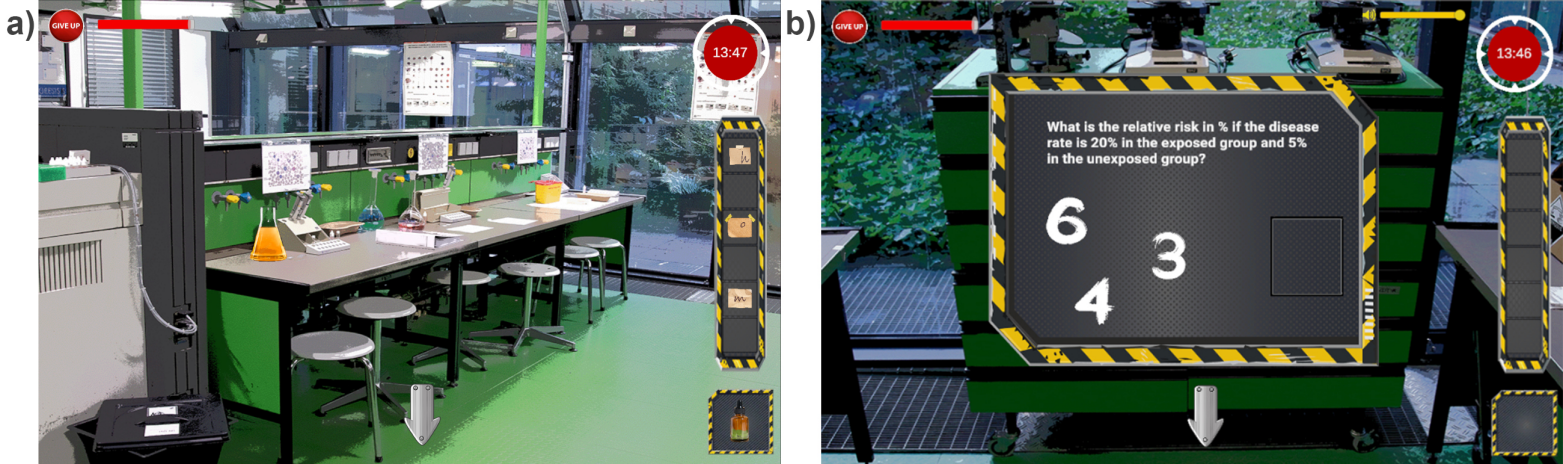
a) Irene Hoffmann laboratory, modeled on the teaching laboratories at the University Hospital RWTH Aachen b) Activities of the game, e.g. puzzles

The OnER was specially developed in the game development engine Unity and the programming language C# and deployed for web usage so that the students could easily access the game and play collaboratively. Dr. Hoffmann’s lab was modeled after the RWTH Aachen University Hospital teaching labs (see figure 1 a (Fig. 1)). The game consisted of eight health science- and location-related (Aachen) activities such as puzzles, quizzes, and riddles (e.g., see figure 1 b (Fig. 1)), which unlocked six different notes with a written letter. These letters are needed for the last puzzle of the game to open the lab door. The student groups should solve all these activities within 15 minutes. 

As soon as the OnER was over, the students and faculty gathered in the Zoom main room to debrief the activity, exchange ideas, and provide feedback. Quantitative data were collected using an online questionnaire with items based on a 5-point Likert scale (1=“strongly disagree”, 5=“strongly agree”). The questionnaire was administered by Mentimeter [https://www.mentimeter.com/de-DE] to assess the acceptance of the OnER. To evaluate the usability of the OnER, the System Usability Scale (SUS) was used [9], [10], [11]. The SUS is a validated and robust 10-item scale asking respondents to rate various aspects of usability on a 5-point Likert scale and indicate their level of agreement. Additionally, qualitative feedback was obtained through an open feedback round to understand the participants’ perspectives on this intervention.

## Results

A total of 35 students (74.28% female) from 11 different countries (Brazil, Czech Republic, Germany, Ireland, Italy, Netherlands, Peru, Romania, Slovenia, United Kingdom, Uruguay) took part in the OnER activity. The students of the program studied Biology, Medicine, Biomedicine, Nutrition Science, Biochemistry or Neuroscience and were on average 22.32±2.52 years old. 

Only one of five groups managed to finish the game on time. 

**Figure 2:**
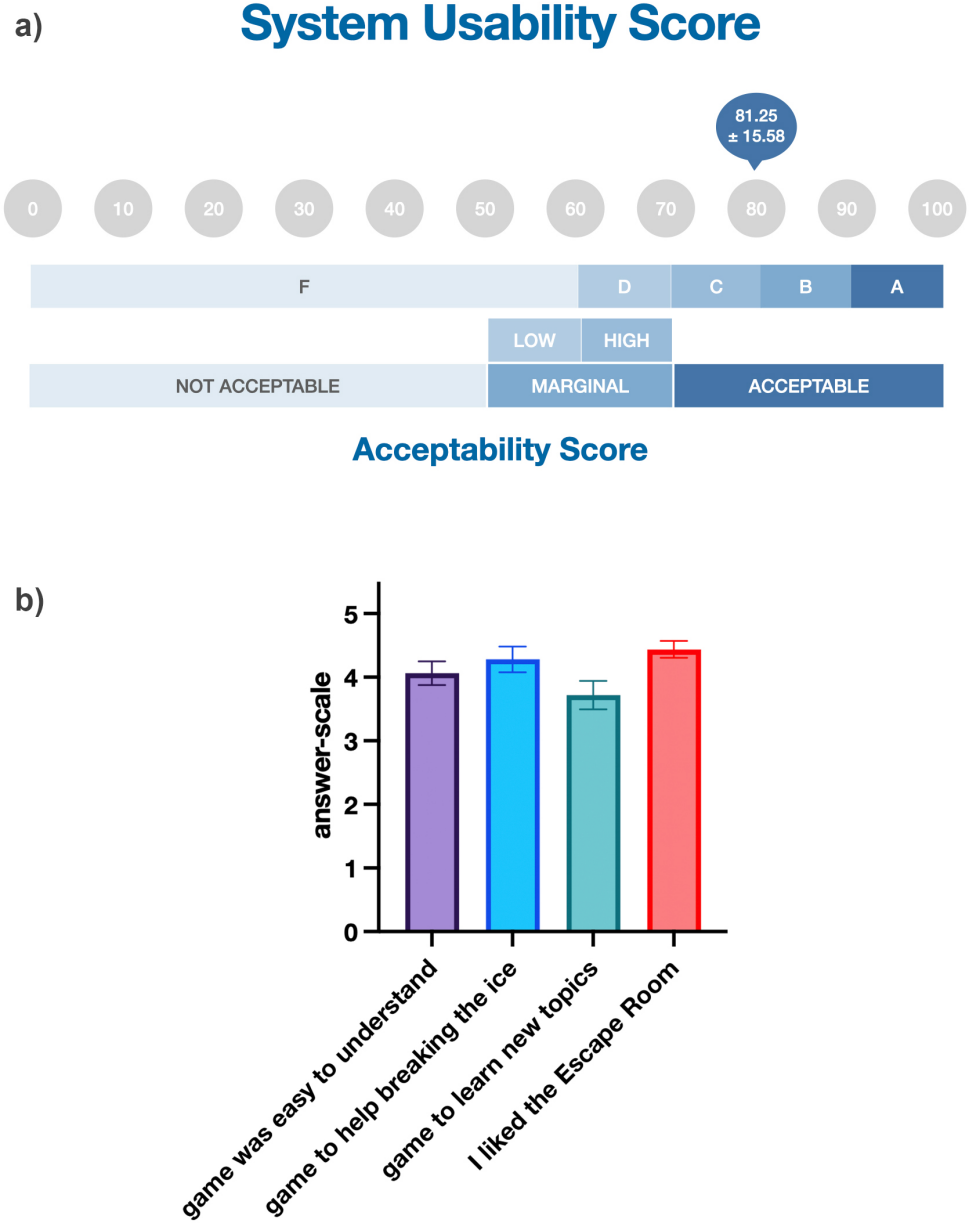
Overview of the results of the students’ re-evaluations

After debriefing, a total of 32 students evaluated the OnER. All students highly appreciated the game (*M*=4.44, ±0.76) and found it easy to understand (*M*=4.06, ±1.05). The SUS was rated in the fourth quartile, and a “B” on a grade scale (M=81.25, ±15.58). The students also agreed that the game is very suitable for use in the classroom to break the ice in small groups (*M*=4.28, ±1.14). Further, students reported that this kind of game could be used in a teaching setting to learn new topics (*M*=3.71, ±1.27). For an overview of the results, see figure 2 (Fig. 2).

In the open-ended feedback, the students reported that they liked the activity and that the idea of using it as an icebreaker was original. One student wrote: "I liked that it was a way to get rid of the awkwardness of meeting my colleagues for the first time". And another: “Great way to break the ice. Also, the questions were not too easy, which adds to the challenge in a positive way!“. Similarly, the topic of the questions was well received: “I like the questions related to the science field.” Some constructive feedback from the students, like new features or bugs, was considered for the next version of the game. 

## Discussion

The escape room’s success as an icebreaker in an international, interdisciplinary, and geographically independent setting may be attributed to its dynamic, interactive nature, offering an innovative alternative to conventional ice-breaking methods. This success aligns well with the broader concept of gamification, which integrates game design elements into non-game contexts, proving particularly effective in engaging today’s students. It has been proven to enhance motivation, increase learner engagement, and foster social interaction within educational settings, making it increasingly popular in medical education [12]. Medical escape rooms exemplify this approach, offering a dynamic platform to teach knowledge, skills, and behaviors in a gamified environment. These escape rooms emphasize collaborative learning through teamwork-driven activities, with clearly defined objectives and immediate feedback, making them a powerful tool for medical education.

Similar to the study by Hursman et al. [8] that investigated virtual interprofessional teams, the teamwork in the current pilot study, in particular, may have led to a cohesive group feeling despite the geographical distance and thus offered a valuable opener for the international program for learning scientific skills in HPE. The integration of HPE research scenarios in the University Hospital in Aachen made it contextually relevant, further enhancing its appeal. Notably, the fact that only one group succeeded in the current OnER could be due to faster and correctly solved tasks or different interactions between participants. A previous study indicates that groups who fail in escape rooms exhibited different interaction patterns compared to successful groups, showing less task-focused behavior and the initial signs of social conflict [13]. As this was not the focus of the current study, a follow-up study should investigate the interactions between students, as well as the individual results of the solved tasks and puzzles, in more detail. Learning analytics, such as tracking of play execution, number of tries and errors made, or video analyses of the group dynamics could be for example analyzed to provide insight into how each team is performing. Although the results are promising, it is essential to consider the scalability of this approach, particularly for larger groups or diverse environments. 

Additionally, access to the technology and resources needed to develop a targeted OnER should be considered, as this could be viewed as a limitation of the described approach.

## Conclusion

This pilot study underscores the potential of OnER as innovative icebreakers in international and interdisciplinary HPE. The positive engagement, teamwork, and contextual relevance outcomes warrant further exploration. Future studies should delve deeper into the long-term benefits of such initiatives, considering diverse student groups and educational contexts.

## Authors’ ORCIDs

- Martin Lemos: [0000-0002-0788-2400]
- Laura Bell: [0000-0002-9425-740X]

## Acknowledgements

We would like to express our gratitude to Sebastian Fedrowitz for his assistance with the software development. We also thank the school for medical-technical laboratory assistants of the RWTH Aachen University Hospital for generously allowing us to use their lab as a setting for the escape room. Our appreciation extends to Uli Heuter for providing the music and sound effects that enhanced the immersive experience.

We also extend our gratitude to the SEMERA coordination team, Eliana Lemos and Anne Hüsgen, for their support in enabling the implementation of this OnER experience within SEMERA and contributing to its practical application and reach.

## Competing interests

The authors declare that they have no competing interests.
